# Differential Glycosylation Levels in Saliva from Patients with Lung or Breast Cancer: A Preliminary Assessment for Early Diagnostic Purposes

**DOI:** 10.3390/metabo11090566

**Published:** 2021-08-24

**Authors:** Andrea Ragusa, Pietrina Romano, Marcello Salvatore Lenucci, Emanuela Civino, Daniele Vergara, Elena Pitotti, Cosimo Neglia, Alessandro Distante, Giampiero Diego Romano, Nicola Di Renzo, Giammarco Surico, Prisco Piscitelli, Michele Maffia

**Affiliations:** 1 Department of Biological and Environmental Sciences and Technologies, Campus Ecotekne, University of Salento, via Monteroni, 73100 Lecce, Italy; marcello.lenucci@unisalento.it (M.S.L.); emanuela.civino@unisalento.it (E.C.); daniele.vergara@unisalento.it (D.V.); 2 CNR-Nanotec, Institute of Nanotechnology, via Monteroni, 73100 Lecce, Italy; 3 Medical Oncology Unit, “Vito Fazzi” Hospital, 73100 Lecce, Italy; info@isbem.it (P.R.); oncologia.polecce@ausl.le.it (G.D.R.); direnzo.ematolecce@gmail.com (N.D.R.); repol@ausl.le.it (G.S.); 4 Local Health Authority ASL Lecce, 73100 Lecce, Italy; proteomica.polecce@ausl.le.it (E.P.); piscitelli@unescochairnapoli.it (P.P.); 5 Euro Mediterranean Scientific Biomedical Institute (ISBEM), 72100 Brindisi, Italy; neglia@isbem.it (C.N.); distante@isbem.it (A.D.)

**Keywords:** HPEAC-PAD, glycoprotein, glycomics, glycosylation, saliva, early diagnosis, lung cancer, breast cancer, fucose, mannose, glucosamine

## Abstract

Glycans play a fundamental role in several biological processes, such as cell–cell adhesion, signaling, and recognition. Similarly, abnormal glycosylation is involved in many pathological processes, among which include tumor growth and progression. Several highly glycosylated proteins found in blood are currently used in clinical practice as cancer biomarkers (e.g., CA125, PSA, and CA19-9). The development of novel non-invasive diagnostic procedures would greatly simplify the screening and discovery of pathologies at an early stage, thus also allowing for simpler treatment and a higher success rate. In this observational study carried out on 68 subjects diagnosed with either breast or lung cancer and 34 healthy volunteers, we hydrolyzed the glycoproteins in saliva and quantified the obtained free sugars (fucose, mannose, galactose, glucosamine, and galactosamine) by using high-performance anion-exchange chromatography with pulsed-amperometric detection (HPAEC-PAD). The glycosidic profiles were compared by using multivariate statistical analysis, showing differential glycosylation patterns among the three categories. Furthermore, Receiver Operating Characteristics (ROC) analysis allowed obtaining a reliable and minimally invasive protocol able to discriminate between healthy and pathological subjects.

## 1. Introduction

Despite a significant reduction in deaths over the last decade, cancer is still one of the major cause of mortality worldwide [1]. Among them, lung cancer (LC) is the deadliest cancer in men and women, while breast cancer (BC) is the second one among women.

The main driver for the steady decline in the overall cancer mortality rates over the past 25 years is the availability of novel treatments and an earlier detection of the disease. Personalized medicine and the discovery of novel biomarkers should help significantly improve the positive trend. In this regard, metabolomics is gaining exponential notoriety, and the exploitation of novel techniques permits deeper insights into cancer related biochemical pathways for improving cancer prognosis and therapy [2,3,4,5,6,7].

It is well-known that glycans, protein-bound carbohydrates, play a fundamental role in several biological processes, such as cell–cell adhesion, signaling, and recognition [8,9]. Nevertheless, they are also involved in many pathological mechanisms, such as inflammation, tumorigenesis, and metastasis in which the normal glycosylation patterns are disrupted because of genetic or acquired disorders [10,11,12].

*N*-glycosylation and *O*-glycosylation events occur in the endoplasmic reticulum (ER) as well as in the Golgi apparatus, generating enormous multiplicity of glycan structures that are part of the human glycome. Several highly glycosylated proteins found in blood are currently used in clinical practice as cancer biomarkers (e.g., CA125, PSA, and CA19-9), indicating the importance of protein glycosylation in cancer and the potential of protein glycosylation analysis in cancer subtype identification and treatment [12,13,14]. Nevertheless, most of these tumor antigens cannot be used alone in the diagnosis of the pathology because of low sensitivity or specificity, thus requiring additional invasive analyses such as biopsies. However, these latter diagnostic techniques are often painful and potentially dangerous for the patient and are time consuming and expensive for the doctor.

In this regard, the advent of liquid biopsy paved the way for screening tumor markers in circulating fluids, i.e., blood, with minimal discomfort for the patient, as well as lower analysis cost. Additionally, liquid biopsy represented a new tool with practical applications not only in prognosis but also in the screening of diseases [15]. Furthermore, progress made from a technological point of view has resulted in increased specificity and sensitivity. However, to date liquid biopsy is not routinely used in clinical practice. From this point of view, the development of a quick and reliable method for the follow-up of tumor progression upon medical treatment of the patient would simplify the process, especially if it requires a non-invasive sampling technique.

Saliva is a readily available substrate that does not bring any harassment to the patient when collected, and it is already proved to be a viable substrate for the detection of several pathologies, such as periodontitis, oral cancer, and cardiovascular diseases [16,17,18,19]. As a complex oral fluid originating from salivary glands, saliva is composed of a plethora of secretory proteins, electrolytes, and other substances known to be age/sex related. This substrate has several advantages over traditional blood withdrawal, for example, the ease of collection, as it does not require puncture. Thus, it is easy, non-invasive, more acceptable for repeated testing, and it can be performed without the need for medical operators or by the patient himself if properly instructed.

Although this approach was already proved to be suitable for metabolomic analysis [20,21], only a few studies investigated the glycoproteome in samples of saliva so far, and most of them were limited to colorimetric assays, such as the Winzler test [22,23]. More recently, Ishikawa and colleagues investigated the use of salivary samples for discriminating oral squamous cell carcinoma from oral lichen planus by salivary metabolomics [24]. In another study, Liu et al. demonstrated that alterations of salivary glycopatterns are related to breast disease [25]. These works already demonstrated that glycomics could be a viable approach for the early detection of cancer diseases by using a non-invasive methodology.

With this in mind, we used saliva samples from patients with either breast or lung cancer and compared their glycomic pattern with that from voluntary healthy subjects. We set-up a quick and reliable methodology for the quantification, through HPEAC-PAD analysis, of simple carbohydrates derived from hydrolyzed glycoproteins in saliva samples. This would allow us to test whether expression levels of carbohydrates in saliva could represent a reliable resource for the early detection of cancer diseases, as well as for the follow up of the pathological process upon pharmacological treatment [26,27,28,29,30].

## 2. Results and Discussion

HPAEC-PAD is a well-known chromatographic technique used for the detection and quantification of simple carbohydrates in various matrices, from biological samples to foods and beverages [31,32]. We already exploited this technique for analyzing the carbohydrate composition of cell wall polymers from plant organs [33,34], agri-food byproducts [35], bacterial exopolysaccharides [36], and fungal communities in insect and human gastrointestinal tract [37]. Here, we decided to investigate its feasibility as a diagnostic tool for the detection of two cancer types when coupled to multivariate statistical analysis. In order to reduce stress for the patient and to amplify the potential number of subjects for screening, we decided to employ this technique for the quantification of carbohydrates hydrolyzed from glycoproteins in saliva samples.

Different methodologies were tested before deciding a final procedure for carrying out the hydrolysis. The hydrolysis was performed both with and without previously centrifuging the saliva sample, but this last step proved to be critical for removing particulate matter and for obtaining clean and reliable data, as suggested by Wishart and colleagues [21,38]. Concentrated hydrochloric acid (HCl, 6 N), sulfuric acid (H_2_SO_4_, 0.5 M), and trifluoroacetic acid (TFA, 2 N) were tested for hydrolyzing the glycoproteins, but no significant differences were noted in the peaks’ area. However, the hydrolysis with TFA produced cleaner samples and less stress on the equipment. Different hydrolysis times (ranging from 1 to 4 h) were also checked in order to determine the minimum reaction time required to completely hydrolyze the samples. Finally, the hydrolysis was conducted at 120 °C for 1.5 h (no significant differences were observed with longer times in the resulting HPLC chromatograms).

**Figure 1:**
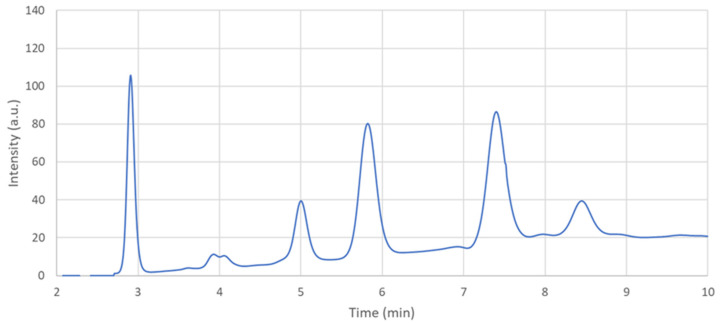
Representative HPAEC-PAD chromatogram of the hydrolyzed carbohydrates from saliva samples. The monitored peaks were the following: fucose (min. 2.9), galactosamine (min. 5.0), glucosamine (min. 5.8), galactose (min. 7.4), glucose (min. 7.9), and mannose (min. 8.4). Samples with a significant glucose peak were discarded because they were hemolyzed.

The chromatographic peaks quantified after hydrolysis corresponded to fucose, galactosamine, glucosamine, galactose, glucose, and mannose after 2.9, 5.0, 5.8, 7.4, 7.9, and 8.4 min, respectively. The glucose signal was observed for identifying hemolyzed samples that were then discharged from the subsequent statistical analysis (Figure 1).

A total of 102 people participated in the study, of which 34 healthy subjects (HC) were used as control, while 68 patients had either breast or lung tumor (38 and 30, respectively) (Table 1).

In order to reduce variability to a minimum, all the people enrolled in the study were chosen with the closest range of age and body mass index (BMI). However, while all subjects had a BMI of about 25–26 kg/m^2^, slightly higher differences were encountered in the average age of the groups, where the healthy volunteers were the youngest (about 46 year old), and the patients with lung cancer were the oldest (about 70 year old). Nevertheless, the difference in age, mainly due to the variances in the development and diagnosis of the two pathologies, did not significantly influence the glycomic pattern within each group, as confirmed by confronting data from patients at different tumor stages.

The majority of the patients with breast cancer had already been previously diagnosed (79%), and about half had been already subjected to clinical surgery (47%) (Table 2). Slightly more than half (53%) tested positive for estrogen receptor markers while a lower portion (39%) tested positive for progesterone receptors markers. Only 34% tested positive for the Her B2 receptor.

Most of the patients with lung cancer had adenocarcinoma (63%), while the remaining either had microcytoma (20%) or squamous carcinoma (3%). Only a few patients were in the first diagnosis while most of them, about 80%, had already been diagnosed between 2014 and 2015, although the full range was from 2010 to 2018. As a consequence, they were already in treatment with chemotherapeutics (mostly based con Pt drugs, i.e., cisplatin and carboplatin, either alone or in combination with antimetabolites, i.e., pemetrexed or topoisomerase II inhibitors, i.e., etoposide), with therapy cycles ranging from 1 to 17 depending on how long they had been in treatment. On the other hand, only a minor percentage of LC patients (about 7%) had been subjected to surgery, e.g., lobectomy.

Saliva samples were collected from patients with either breast or lung cancer (n = 38 and 30, respectively), as well as from healthy volunteers (n = 34) used as control. The samples were hydrolyzed for 1 h with TFA at 120 °C. Sulfuric acid and different hydrolysis times were also tested but TFA produced a cleaner product, while increasing the reaction time did not result in an improved yield. The hydrolyzed monomeric sugars contained in the saliva were then analyzed by HPAEC-PAD and the peaks of fucose, galactosamine, galactose, glucosamine, and mannose were quantified.

Mean and median concentrations, as well as standard errors and deviations, obtained for the different carbohydrates are reported in Table 3.

**Figure 2:**
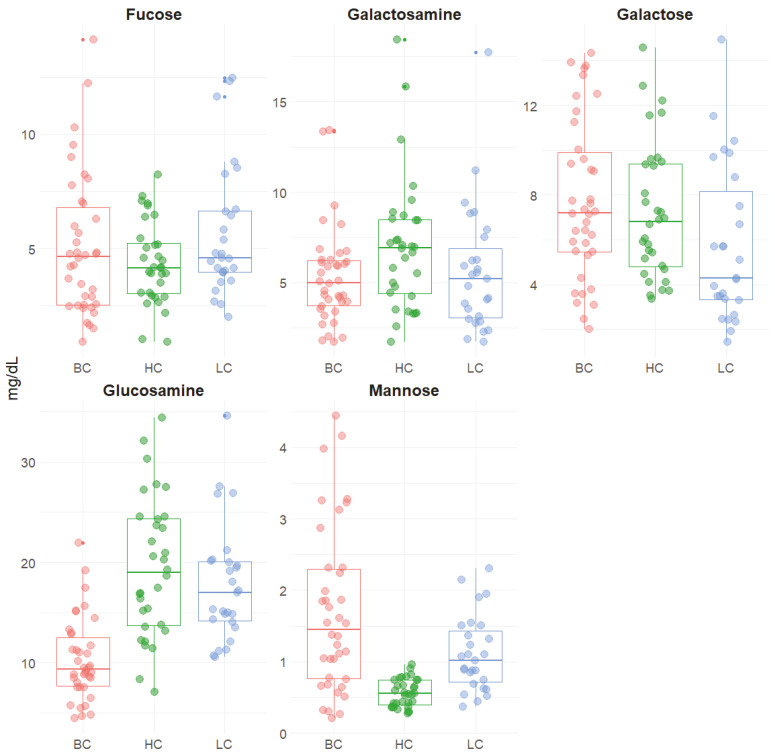
Dot plots reporting the concentrations of the hydrolyzed carbohydrates from saliva samples of patients with breast cancer (BC), lung cancer (LC), or healthy controls (HC).

Figure 2 shows the scatter plot distribution of the hydrolyzed carbohydrates in the saliva from patients with either breast cancer or lung cancer and that in healthy patients.

It can be observed that both fucose and mannose are overexpressed in pathological samples, while galactosamine is underexpressed. Differences were also observed between the two pathological samples. In particular, galactose was underexpressed in LC samples, while glucosamine was underexpressed in BC ones.

This is quite in agreement with data from Ruhaak and colleagues who conducted a proteomic analysis on lung adenocarcinoma tissues [39]. They found that the levels of several oligomannose-type glycans were upregulated in adenocarcinoma tissues, while fully galactosylated glycans were decreased, whereas low-galactosaylated or non-galactosylated glycans mostly with fucose were increased.

Interestingly, mannose concentrations were shown to possess quite a narrow distribution in healthy subjects, while they presented a much wider range in pathological samples, especially in those from patients with breast cancer.

Our results show how fucose levels are higher in both cancer samples than controls. This result is in line with data in the literature citing highly fucosylated protein presence in the serum sample of patients affected by both small and non-small lung cancer cells [40,41], where fucosylation can be additionally used as a prognostic tool for cancer progression [42]. The importance of aberrant fucosylation in breast cancer has been less investigated, although several studies also showed elevated fucosylation of specific serum proteins in breast cancer patients (refer to [43] for a review). Our results are also in agreement with studies related to mannose expression levels in cancer samples, as known from the literature for both lung and breast cancer [44,45]. Interestingly, Fang and colleagues were also able to distinguish, through a barcode matching protocol by using mass analysis, among the four different types of breast cancer by analyzing the serum of patients [45].

Although the overexpression of both *O*-linked and *N*-linked glycosylation patterns is more common in cancer progression, decreased *O*-glycosylation has been also reported in breast cancer, resulting in mucin-type tumor-associated antigens [46]. This phenomenon might explain why lower amounts of glucosamine were found in saliva samples from breast cancer. Nevertheless, this finding deserves further investigation because this carbohydrate is usually found in higher amounts in cancer patients, although in different matrices, i.e., tissue.

Compared to control samples, Balog et al. found lower glucosamine levels in colorectal cancer tissues, together with a higher expression of sulfated and paucimannosidic glycans as well as glycans containing sialylated Lewis epitopes [47].

Differences with already reported studies might be due to the different substrates employed. In fact, the differential expression of glycoproteins according to the analyzed matrix has been already been observed by Takakura et al., who observed excess of monosyalated and fucosylated glycans in the membrane proteins from fetal lung fibroblasts, while desialylated and afucosylated glycans were predominant in serum proteins [48].

### 2.1. Statistical Analysis

In order to check statistically significant differences among carbohydrates in each individual group, a *t*-test was performed (Table 4). As expected, fucose was significantly overexpressed in both pathologies compared to the healthy subjects. This is not surprising considering that the aberrant fucosylation of glycans is a well-known post-translational modification that has been observed in a variety of tumors, including breast and lung cancers in blood as well as in tissues [42,49,50]. This result confirms that fucose metabolism is also altered in other human fluids, i.e., saliva. Similar results have already been reported in saliva but at a less quantitative level as less precise methodologies were utilized, such as the colorimetric Winzler assay. On the other hand, no differences were noted between the two pathologies, both averaging over 5 mg/dL.

Similarly, mannose was shown to be significantly overexpressed in both pathological samples, although in this case some differences were also noted between the two diseases, with the BC samples showing slightly higher concentrations.

Interestingly, BC samples contained statistically different levels of glucosamine and galactose compared to both controls and LC samples. On the other hand, the distribution of galactosamine was quite narrow among all samples.

**Figure 3:**
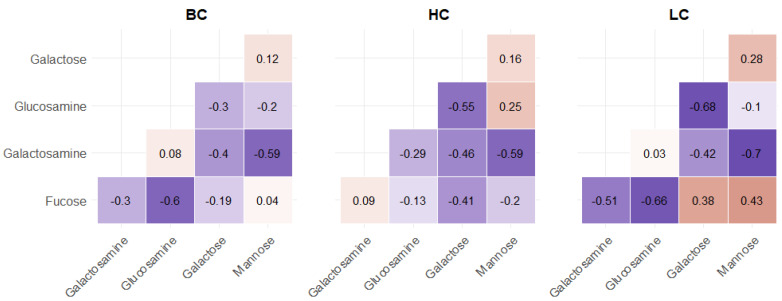
Matrix of correlation plots among the hydrolyzed carbohydrates in each group of samples.

Analysis of the relationship among the levels of the carbohydrates in the different classes showed a similar trend. Nevertheless, some differences in the correlations were observed, especially in the LC samples (Figure 3).

Interestingly, the stronger correlations had all negative values. A significant negative correlation was always observed between galactosamine and mannose (−0.6), and it was even more pronounced in the LC samples (−0.7). A quite strong negative correlation (between −0.6 and −0.7) was also observed between fucose and glucosamine but only in the pathological samples.

Nevertheless, in the BC samples, only the correlations between fucose and glucosamine and between galactosamine and either galactose or mannose resulted significant by looking at the corresponding *p*-values. Correlations with galactosamine were also statistically significant in LC and HC samples, while differences were observed in the fucose behavior. In fact, it showed moderate (−0.41) correlation with galactose in HC samples. On the other hand, an opposite trend was observed in LC samples, where fucose significantly correlated with galactosamine, glucosamine, and mannose (−0.51, −0.66, and 0.43, respectively). Interestingly, the positive relationship between fucose and mannose, two well-known carbohydrates overexpressed in several tumor types, was relevant only in patients with lung cancer but not in those with breast cancer.

**Figure 4:**
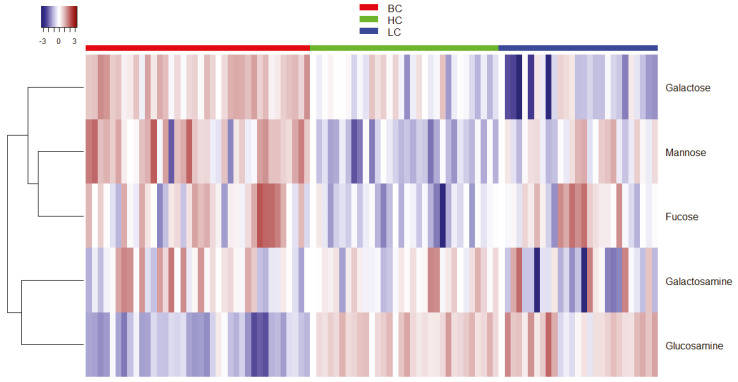
Heatmap analysis of the hydrolyzed carbohydrates (BC = red, HC = green, and LC = blue).

In order to better highlight the abundance of the hydrolyzed carbohydrates according to the type of disease, a heatmap was built, confirming the higher amount of galactose and mannose in the BC samples (Figure 4).

On the other hand, glucosamine was underexpressed in the patients with breast cancer compared to those with lung cancer or healthy subjects, while galactose was underexpressed in the LC samples. As expected, mannose and, even more, fucose were overexpressed in both pathological samples.

**Figure 5:**
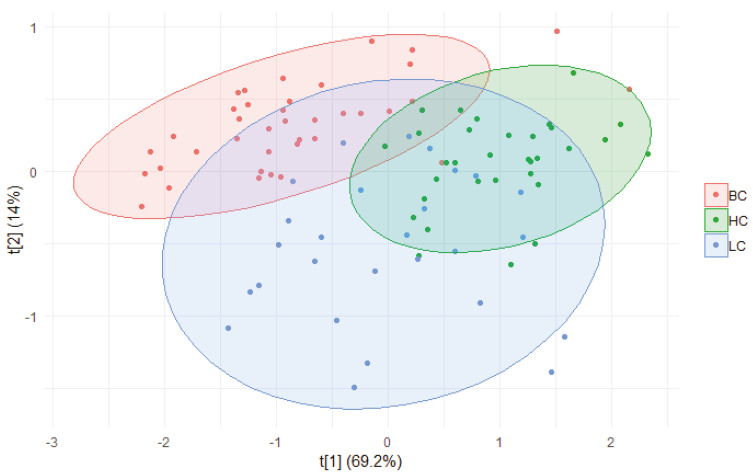
Principal component analysis (PC1 vs. PC2) score scatterplot of the hydrolyzed carbohydrates from the BC, LC, and HC samples.

In order to maximize variance and appreciate the differences among the classes, principal component analysis (PCA) was performed. The first two components alone were able to explain 83.2% of the population. However, although both pathological samples and healthy controls grouped quite nicely and some separation could be observed, a significant overlap was noted (Figure 5).

Nevertheless, good separation was noted between the BC and HC samples, especially along the PC1, while the LC samples were more spread along both components.

**Figure 6:**
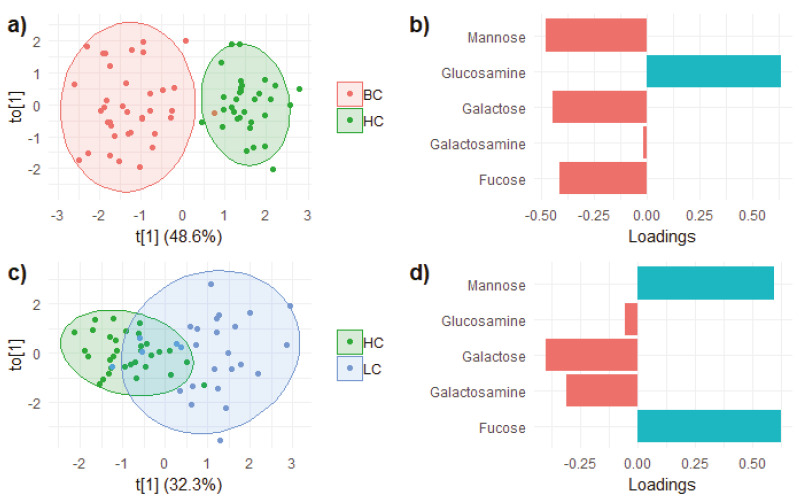
OPLS-DA score scatterplot of the hydrolyzed carbohydrates in (**a**) BC vs. HC and (**c**) LC vs. HC samples. The corresponding loading plots are reported in (**b**,**d**), respectively.

In trying to improve separation and to enhance the differences among groups, the supervised orthogonal partial least squares discriminant analysis (OPLS-DA) was then performed independently between each pathological group and the healthy controls (Figure 6).

As expected, separation was improved in both cases, although only BC samples gathered into a group that did not overlap with that of the HC one. Again, the main variables responsible for the separation were mannose and fucose present in higher concentrations in the pathological samples and glucosamine which, instead, was less concentrated in the BC samples. Underexpression of galactose and galactosamine in the LC samples and overexpression of galactose in the BC samples were also relevant for the separation of the two pathological groups from the HC one, as already suggested by the previous analyses.

### 2.2. ROC Analysis

Receiver Operating Characteristics (ROC) analysis, a well-established machine learning technique generating a model able to distinguish between different classes, already proved to be a valuable tool with enormous clinical potential in diagnosing several pathologies [51,52,53,54]. Nevertheless, this methodology is usually applied to samples obtained with invasive techniques, such as intravenous blood collection or biopsy. On the other hand, exploitation of minimally invasive and quick tests would allow easy screening of a large number of subjects and can be used as non-specific predictors of a pathology. In this regard, sputum collection represents a non-invasive procedure for this type of analysis.

Thus, we investigated the possibility of creating a ROC-based model for predicting the possibility of having breast or lung cancer according to the data obtained by analyzing the glycomic profile in saliva samples. This would bring a step forward for the creation of a simple and non-invasive preliminary screening test that, if positive, would require additional analysis before confirming the pathology anyway.

**Figure 7:**
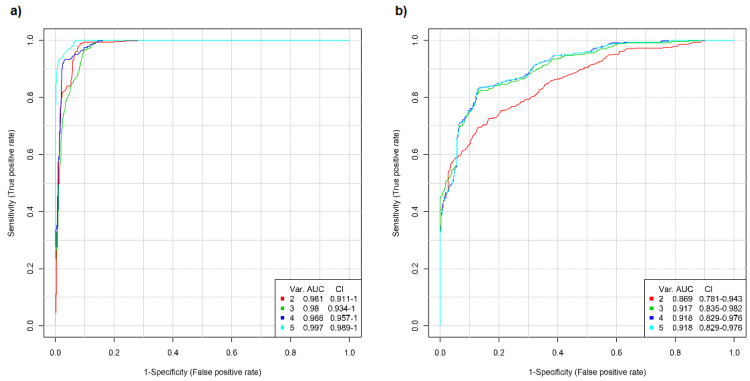
Multivariate ROC curves obtained by comparing the glycomic profile of the (**a**) breast cancer and (**b**) lung cancer patients to the glycomic profile of healthy subjects.

As expected, the use of a multivariate model yielded much better results compared to the ROC curve analysis based on individual carbohydrates (data not shown). The partial least squares discriminant analysis (PLS-DA) multivariate algorithm was used to generate the ROC curves, obtaining very good results in both cases when comparing the pathological profiles to that of the healthy subjects (Figure 7).

Excellent prediction was obtained for the screening of breast cancer (Figure 7a). The use of only two variables, i.e., glucosamine and mannose, already yielded an area under the curve (AUC) of 0.98 with a confidence interval (CI) of 0.91–1, and the inclusion of an additional carbohydrate, i.e., galactose, did not improve the AUC but raised the CI to 0.93–1. On the other hand, the addition of fucose increased the AUC to almost 0.99, with a CI of 0.96–1. Nevertheless, inclusion of all five carbohydrates provided a multivariate model with a stunning AUC of >0.99 and a CI of almost 0.99 to 1.

A slightly lower but still very good AUC was noted for patients with lung cancer, with AUC ranging from 0.87 to 0.92 (Figure 7b). In this case mannose and fucose yielded a model with an AUC of almost 0.87 and a CI of 0.78–0.94. The addition of galactose increased the AUC to almost 0.92 (CI of 0.84–0.98), but no significant improvements were noted when including galactosamine and glucosamine.

Although these results are already encouraging, screening of additional patients, even suffering from different tumors, and control subjects would allow the improvement of sensitivity and specificity for both models. Furthermore, since the sampling uses non-invasive methodology, it could be used as a preliminary screening of unknown subjects and, in the case of a potential positive result, suggest a more invasive and detailed analysis on blood or tissue samples.

## 3. Materials and Methods

### 3.1. Cohort Recruitment and Sample Preparation

#### 3.1.1. Patients’ Recruitment

After the approval (No. 3, 09/03/2017) of the Ethical Committee of Local Health Authority ASL Lecce (Italy) in cooperation with the University of Salento and the Euro Mediterranean Scientific Biomedical Institute (ISBEM), the enrolment at the outpatients’ ambulatories of “Vito Fazzi” Hospital (Division of Oncology, Lecce, Italy) was conducted between June and December 2017. A total of 68 consecutive patients with first diagnosis of breast cancer (n = 38) or lung cancer (n = 30, including mesothelioma), naive to chemotherapy or treatments with biological drugs, were enrolled. A total of 34 age-matched healthy subjects were consecutively recruited among blood donors at the Transfusion Service of P. O. “Vito Fazzi” in Lecce (Italy) as the control group. The recruitment phase duration was set at 1 year. The calculation of the sample size was carried out in the hypothesis of a lower limit of the Confidence Interval (CI) set at 0.95, considering alpha equal to 0.05 and a power of 90%. Personnel from local health authority ASL Lecce was responsible for the enrollment and the collection of signed informed consents, as well as anamnestic questionnaires specifically developed to assess inclusion and exclusion criteria. The study has been carried out according to the Helsinki declaration and good clinical practice. Privacy of the enrolled subjects was guaranteed by treating data anonymously based on numerical codes assigned to each patient or control.

#### 3.1.2. Inclusion and Exclusion Criteria

Male and female subjects, aged >18 years old, with a BMI of about 25–26 kg/m^2^, and with an established clinical diagnosis of either breast or lung cancer (including mesothelioma) were eligible. Pregnant patients as well as patients with previous history of other malignancies or in the terminal stage (expected less than 4 weeks old) were excluded from the enrollment. Patients who had conditions that might have potentially interfered from a metabolic point of view were excluded. Patients simultaneously suffering from liver cirrhosis, gastric ulcer, diabetes mellitus, and periodontitis were also excluded from the study.

#### 3.1.3. Saliva Samples Collection

All samples were collected early in the morning. Patients and healthy donors accessing the recruiting center considered eligible for the study and that were fasting from the night before were asked to provide sputum (about 3 mL) in a sterilized plastic vial that was immediately transferred to the division of Proteomics at the same “Vito Fazzi” Hospital. After centrifugation at 1500 rcf for 10 min to precipitate the particulate, the supernatant was aliquoted in sterilized screw cap plastic vials (0.4 mL of saliva sample each) and stored at −80 °C until hydrolysis.

### 3.2. Hydrolysis Procedure

Saliva samples (0.4 mL) were thawed at room temperature and 2 N TFA (0.1 mL) added. The sample was placed in an autoclave for 1 h at 120 °C, after which the solvent was evaporated with a SpeedVac concentrator. Double distilled water (1 mL) was added to the residue. An aliquot (0.5 mL) was withdrawn with a syringe, filtered (MWCO 3000 Da), and injected into the HPLC for analysis.

### 3.3. HPAEC-PAD Analysis

The analysis of the hydrolyzed samples was performed with a High-Performance Anion Exchange Liquid Chromatography with Pulsed Amperometric Detector (HPAEC-PAD) Dionex system composed of LC25 Chromatography Oven, GP50 Gradient Pump, AD25 Absorbance Detector, ED50 Electrochemical Detector, and equipped with a Column CarboPac PA10. The flow rate was maintained at 1 mL/min at room temperature. The injection volume was 20 mL. HPLC-grade H_2_O was used as eluent A, while 0.05 and 0.8 M NaOH were used as eluent B and C, respectively. The gradient system was as follows: 0–20 min, 60% A and 40% B; 20–27 min, 75% A and 25% C; 27–31 min, 100% C; 31–42 min, 60% A and 40% B. Standard solutions of fucose, galactosamine, glucosamine, galactose, glucose, and mannose were prepared and analyzed at different concentrations in order to obtain a calibration curve for each analyte. Limit of detection (LOD) and limit of quantification (LOQ) were 0.003 and 0.009 µg/mL, respectively. Each sample was analyzed in triplicate and quantified by interpolation of the area of the peak. Chromeleon Client software v 6.80 was used to process the raw chromatograms. A data table with the concentrations of the analytes for each sample was finally obtained.

### 3.4. Statistical Analysis

Statistical analysis was performed with *R* (version 3.5.2). The descriptive results are expressed as the mean ± standard deviation, unless otherwise stated. Normality of the variables was checked by the Shapiro–Wilk normality test, while homogeneity was checked by using the F-test. Samples were normalized by sum, and the data log was transformed and mean centered before statistical analysis. Statistical difference between the values were assessed using the Student’s *t*-test (a value of 5%).

Pearson’s correlation was used to check linear relationships between normally distributed numerical variables, while Spearman’s rank correlation was used in all other cases. The *p*-values were adjusted for multiple comparison by controlling the false discovery rate (FDR, proportion of false positives among the metabolites called significant) at a 5% threshold.

PCA and OPLS-DA were performed by using the *ropls* R package [55]. The goodness of the model was assessed by the R^2^ coefficient, while its predictive ability was assessed by the Q^2^ coefficient by performing a 7-fold cross-validation. Significance of the model was assessed through 1000 random permutations (*p*-value < 0.001).

Univariate and multivariate ROC analyses were performed using the *MetaboAnalystR 2.0* R package [56]. PLS-DA was used to classify the models using 2 latent variables. Two-thirds of the population was used as training set, while the remaining one-third was as the validation set. A cut-off of 0.5 was set in order to calculate the ROC curve. The ROC curves were generated through Monte-Carlo cross validation (MCCV) by using balanced subsampling. In each MCCV, feature importance was evaluated by using part of the samples, and the remaining samples are used to validate the models created with the first step. The top-ranking features are used to build the biomarker classification models. This is repeated several times in order to calculate the performance and confidence intervals of each model.

## 4. Conclusions

The early diagnosis of cancer is a major target in current research medicine because it can completely change the outcome of the disease. In this regard, saliva can represent a perfect matrix for non-invasive sampling and screening. Glycomics is gaining ever increasing importance because of its fundamental role in several physiological and pathological conditions. Similarly, aberrant glycosylation patterns have been already observed in tissue, blood, and saliva samples from patients with various types of cancer.

With this aim in mind, we developed a simple and quick protocol by HPAEC-PAD analysis able to quantify major glucosides, i.e., fucose, mannose, glucosamine, galactosamine, and galactose, in hydrolyzed glycoproteins from saliva samples. Aberrant concentrations of fucose and mannose were observed in patients with either breast or lung cancer, as compared to healthy subjects. Furthermore, the exploitation of multivariate statistical analysis techniques allowed obtaining a model able to distinguish between the two pathologies and to predict, with very good sensitivity and specificity, the association with each of them by looking at the glycomic salivary profile.

The exploitation of novel techniques for discriminating between healthy subjects and tumor patients has been already reported in the literature by analyzing blood or tissue samples. Nevertheless, our proof-of-principle approach not only demonstrated the ability to discriminate between tumor patients and healthy persons but also between subjects with different tumor types by exploiting a minimally invasive sampling technique, such as sputum.

Our results show how glycoprotein levels in saliva samples from patients affected either from breast or lung cancer diseases are differentially expressed compared to the healthy control. In particular, both fucose and mannose were overexpressed in pathological samples. On the other hand, the other hydrolyzed carbohydrates showed a different pattern apparently related to the specific disease.

Our approach provides a simple, quick, and non-invasive technique that might result in the definition of a reliable tool that could be exploited in early diagnosis, as well as in the follow up of the pathology. A wider audience will hopefully confirm the potentiality of this quick and non-invasive approach. Similarly, additional and more detailed studies investigating the differential glycosylation patterns characterizing cancer patients and healthy controls will help to enhance the sensitivity and the accuracy of glycoproteins’ characterization as cancer biomarkers.

## Figures and Tables

**Table 1:** Summary data of the participants in the study.

	BC (n = 38)	HC (n = 34)	LC (n = 30)
Gender ^1^ (n, %)	0, 0%	16, 42%	22, 58%
Age (years)	54.2 ±13.0	46.2 ± 10.8	69.8 ± 10.3
BMI (kg/m^2^)	26.6 ± 5.0	25.0 ± 3.1	25.2 ± 4.3

^1^ Expressed as number and percentage of males.

**Table 2:** Summary data of the BC and LC patients.

Cancer Type	Clinical Information ^1^	Yes	No	N.A.
BC	Surgery (n, %)	18, 47%	12, 32%	6, 16%
	First diagnosis (n, %)	5, 13%	30, 79%	1, 3%
LC	Surgery (n, %)	7, 23%	20, 67%	3, 10%
	First diagnosis (n, %)	2, 7%	25, 83%	3, 10%

^1^ Expressed as number and percentage of males.

**Table 3:** Concentrations in mg/dL of the hydrolyzed carbohydrates as determined by HPAEC-PAD.

	Fucose			Galactosamine			Galactose			Glucosamine			Mannose		
	BC	HC	LC	BC	HC	LC	BC	HC	LC	BC	HC	LC	BC	HC	LC
Mean	5.07	4.37	5.70	5.32	6.99	5.63	7.79	7.23	5.73	10.30	19.47	17.81	1.69	0.56	1.11
Median	4.65	4.16	4.58	5.00	6.93	5.21	7.21	6.80	4.30	9.36	18.97	16.97	1.45	0.55	1.02
sd ^1^	3.09	1.81	2.90	2.65	3.66	3.49	3.56	3.03	3.48	4.07	6.94	5.83	1.15	0.19	0.53
se ^2^	0.50	0.32	0.56	0.43	0.65	0.67	0.58	0.54	0.67	0.66	1.23	1.12	0.19	0.03	0.10

^1^ Standard deviation. ^2^ Standard error.

**Table 4:** Results of the *t*-test between pairs of groups performed on the hydrolyzed carbohydrates. * represents a *p*-value < 0.05, ** a *p*-value < 0.01, and **** a *p*-value < 0.001, ns = not significant.

	Group 1	Group 2	*p*-Value	Adjusted *p*-Value	Significance Level
Fucose	BC	HC	3.35 × 10^−7^	1.00 × 10^−6^	****
	BC	LC	0.77783	0.78	ns
	HC	LC	8.94 × 10^−6^	1.80 × 10^−5^	****
Galactosamine	BC	HC	0.873575	0.87	ns
	BC	LC	0.018677	0.056	*
	HC	LC	0.019561	0.056	*
Glucosamine	BC	HC	1.21 × 10^−23^	3.60 × 10^−23^	****
	BC	LC	1.68 × 10^−15^	3.40 × 10^−15^	****
	HC	LC	0.773069	0.77	ns
Galactose	BC	HC	1.49 × 10^−8^	4.50 × 10^−8^	****
	BC	LC	1.87 × 10^−8^	4.50 × 10^−8^	****
	HC	LC	0.002665	0.0027	**
Mannose	BC	HC	6.47 × 10^−13^	1.90 × 10^−12^	****
	BC	LC	0.002107	0.0021	**
	HC	LC	9.65 × 10^−7^	1.90 × 10^−6^	****

## Data Availability

Raw data are available upon request from the corresponding authors. The data are property of the hospital “Vito Fazzi” and also contain personal data of the patients.
